# Ambiguities in Washington State hospital policies, irrespective of Catholic affiliation, regarding abortion and contraception service provision

**DOI:** 10.1186/s12978-018-0621-5

**Published:** 2018-10-19

**Authors:** Hilary M Schwandt, Bethany Sparkle, Moriah Post-Kinney

**Affiliations:** Fairhaven College, Western Washington University, 516 High Street, MS 9118, Bellingham, WA 98225 USA

**Keywords:** Washington State, Reproductive health policy, Hospital, Catholic-affiliated hospital, Abortion, Contraception

## Abstract

**Background:**

In 2014, the governor of Washington State mandated that all hospitals publically post a reproductive health policy amidst concerns about the lack of clarity among the public how hospitals handled various aspects of reproductive health care.

**Methods:**

The objective of this study is to assess the clarity of abortion and contraception service provision in the hospital reproductive health policies for the public in Washington State. All Washington State hospital reproductive health policies (*n* = 88) were analyzed in 2016 using content analysis. Results were stratified by Catholic religious affiliation of the hospital.

**Results:**

There were more similarities than differences between the non-Catholic and Catholic hospital reproductive health policies; however, there were a few differences. Non-Catholic hospitals were more likely than Catholic hospitals to use legal language (except for emergency contraception), include conscientious clause for providers (44% vs. 0%), and were less likely to specify that emergency contraception use was available for sexual assault victims only (16% vs 54%). Most hospital reproductive health policies, regardless of Catholic affiliation, provided more confusion than clarity in terms of abortion and contraception service provision.

**Conclusions:**

The impact of Catholic, and non-Catholic, affiliated hospital care on patients who need abortion and contraceptive services is concerning. Given the difficulties in meeting the goals of increased transparency for the public through hospital policy language, the government should instead mandate hospitals use a standardized checklist. Additionally, patients are in dire need of positive rights to information about and services to avoid the potential gap in care that the negative rights afforded to providers and facilities to opt-out of providing abortion and contraceptive services have created.

## Plain English summary

In 2014, the governor of Washington State mandated that all Washington State hospitals publically post their hospital’s reproductive health policy. The public was concerned about the lack of clarity on how hospitals handled various aspects of reproductive health care – especially given the high, and growing, number of Catholic affiliated hospitals in the state. The objective of this study is to assess the clarity of abortion and contraception service provision in those hospital reproductive health policies for the public in Washington State. All Washington State hospital reproductive health policies (*n* = 88) were analyzed in 2016. Results were examined according to Catholic affiliation of the hospital. Most hospital reproductive health policies, regardless of Catholic affiliation, provided more confusion than clarity in terms of abortion and contraceptive service provision. The impact of Catholic, and non-Catholic, affiliated hospital care for abortion and contraceptive service provision is concerning. Given the difficulties in meeting the goals of increased abortion and contraceptive healthcare guideline transparency for the public through hospital policy language, the authors recommend use of a standardized checklist by hospitals to convey abortion and contraceptive service provision. The authors also recommend that patients have more positive rights to information and services to counter balance the negative rights afforded to providers to op-out of providing abortion and contraceptive services.

## Background

Over the past fifteen years the number of Catholic hospitals in the US has grown by 22%. Ten of the top 25 health systems in the US are Catholic, furthermore, one in six beds are now in Catholic hospitals [1]. The historically non-religious Washington State, deemed a state with a supportive abortion policy environment [2], has been particularly impacted by this shift, where 34% of the hospitals and 40% of the hospital beds are located in Catholic affiliated hospitals – greater than the national average [1]. This trend of increasing hospital care falling under Catholic jurisdiction is predicted to continue [3].

Reproductive health is an area of concern with Catholic dominance in healthcare. Staff in Catholic health institutions are required to abide by what are known as the “Ethical and Religious Directives for Catholic Health Care Services” (ERDs) [4]. The ERDs declare abortion is allowed only if there is no alternative option to save the mother’s life, as well as a ban on all sterilizations and contraception used solely to prevent conception. The interpretation of the ERDs, and therefore care provided, differs by hospital and local bishop. This has been shown to be particularly relevant after hospital mergers [5]. In contrast to the ERDs, the American College of Obstetricians suggests abortions be obtained in a timely and unbiased manner [6]. Physicians at Catholic hospitals report hospital ethics committees deciding against their best judgement in the case of a fetal heart beat – despite the fact that the fetus is nonviable, or continuation of the pregnancy is life-threatening [7]. As a result of these interferences in reproductive medical care, there have been a few high-profile cases of mis-management of miscarriages due to decisions impacted by the ERDs [1].

Obstetrician-gynecologists report disagreeing with a ban on sterilization [8]. Female sterilization is the safest for women immediately following a delivery, for either vaginal or Caesarean births [9]. In general, restrictions on contraceptive access, including sterilization, at Catholic hospitals have led to an increase in subsequent pregnancies [10]. Lack of access to timely abortion and contraceptive services can place undue hardships on patients with less financial resources [5, 8].

Women are often unaware that the hospital they attend for reproductive health care is Catholic. A recent study found that women indicating they attend a Catholic hospital for reproductive health care were nearly six times more likely to misidentify the hospital as religiously affiliated as compared to women who attend non-Catholic hospitals [11]. Women are also unaware that Catholic affiliation of hospitals affects the contraception and abortion care they can receive. A study in Colorado examined reproductive aged women’s perceptions about contraceptive and abortive care provision by hospital type, Catholic affiliated and secular, and found no difference in women’s expectations of contraceptive and abortive service provision at the two facilities except for advice about natural family planning methods [12].The ERDs are publicly available online for those who know to look for them; however, within hospitals they are provided only as an internal document and are not posted publicly on hospital websites. As a result, women are likely unaware of how their reproductive health care is limited when attending a Catholic affiliated hospital, nor will they be informed of these constraints while services are being offered.

Research shows restrictions on abortion and contraceptive service provision is not limited to Catholic affiliated health facilities. The number of facilities, and providers, offering abortion services in the USA [13], and specifically Washington State [14] has been decreasing. Perhaps in a correlated way, the abortion rate has been decreasing while the fertility rate has been increasing in Washington State [15]. Research on emergency contraception (EC) availability in hospital emergency departments in the USA found a similarly high percentage of Catholic and non-Catholic hospitals did not have EC available – and among those without EC available, only half offered referrals for EC. Unfortunately, most of the referrals were ineffective upon follow-up. Slightly more Catholic hospitals said they would provide EC only in cases of sexual assault than non-Catholic hospitals, but in both cases less than a quarter of the sample fell into this category [16]. Abortion and contraceptive services may be constrained at all types of hospitals – not just Catholic ones.

In January of 2014, the Washington State Department of Health via a mandate from the governor, required all Washington State hospitals to publicly post on the Department of Health website their current Reproductive Health policies (WAC 246–320-141) with the aim of increasing the public’s understanding of each hospital’s policy on their specific provision of reproductive health care. This study aims to discern whether the reproductive health policies posted provide clarity to the public about the provision of reproductive health care affected by the ERDs, specifically abortion and contraception, at hospitals in Washington State and whether clarity about service provision differs by Catholic affiliation of the hospital.

## Methods

The reproductive health policies for all hospitals providing reproductive health care in Washington State were analyzed for clarity using content analysis on selected reproductive health areas impacted by the ERDs from May to July of 2016 [17]. The reproductive health areas analyzed in the policies: reproductive health, abortion, contraception, and emergency contraception, were selected based upon divergence in recommended care between Catholic doctrine via the ERDs and best health practices as outlined by medical experts.

Hospital reproductive health policy text was coded as reproductive health when language existed in the policy that explicitly stated reproductive health or was relating to the broad topic of reproductive health (1 = reproductive health theme present, 0 = reproductive health theme absent), this did not include specific types of reproductive health service provision. If the reproductive health theme was indicated as present, it was further analyzed whether reproductive health was framed in terms of legal language or not (1 = reproductive health in terms of the law, 0 = reproductive health not in terms of the law).

For abortion – any text relating to communication about, or services provided related to a direct or indirect termination of pregnancy, as well as pre-care and immediate follow-up care, was coded as abortion (1 = abortion theme present, 0 = abortion theme absent). If abortion was coded as present in a policy, further subcategories of abortion were assessed. The subcategories included abortion provision (1 = abortions provided, 0 = abortions not provided); abortion type provided - whether medical and elective abortions were provided (1), provision extended to medical abortion only (2), referrals provided (3), and whether what types of abortions provided were unclear (4); whether provision of abortion was presented in terms of the law (1 = abortion legal language, 0 = no abortion legal language); and whether the option provided for health care providers to opt out of abortion provision was included (1 = provider opt out option for abortion included, 0 = provider opt out option for abortion not included).

Any mention or discussion of provision of methods designed for the prevention of pregnancy were coded as contraception (1 = contraceptive services mentioned, 0 = contraceptive services not mentioned). If contraceptive services was coded as mentioned in a policy, whether contraceptive services were provided (1 = contraceptive services provided, 0 = contraceptive services not provided), contraceptive service provision was clearly stated or not (1 = clear contraceptive service provision, 0 = unclear contraceptive service provision), and whether contraceptive service provision was presented in terms of legal language (1 = contraception provision described in terms of legal language, 0 = contraceptive provision not described in terms of legal language) was also assessed as subcategories of contraceptive services.

For emergency contraception – any policy language about the discussion and provision of emergency contraception was coded as such (1 = emergency contraception included; 0 = emergency contraception not included). Among policies that included any mention of emergency contraception, two subcategories were also assessed – (1) whether emergency contraception provision was explained in terms of legal language (1 = emergency contraception provision in terms of legal language, 0 = emergency contraception not in terms of legal language); and (2) whether the provision of emergency contraception was mentioned in reference to sexual assault patients (1 = emergency contraception in reference to sexual assault, 0 = emergency contraception not in reference to sexual assault). All authors independently coded all policies, all inconsistencies were discussed and final decisions were made collaboratively. Direct quotes were collected to illustrate findings for each code category.

Clarity was assessed through multiple mechanisms. First, through inclusion of the term, or language that implied the term. Second through policy language indicating unambiguously directly related hospital policy and associated procedures (excluding reproductive health). Ultimately, a clear policy would include mention of each of the reproductive health terms, or language indicating the same topic as the terms, as well as language to explicitly detail the hospital procedure in terms of each code. Each hospital reproductive health policy could be coded as including all of the reproductive health areas – and associated details about policy and provision, some, or none of them.

The type of hospital organization (individual or conglomerate) and length of policy (in terms of page length) were recorded. A reproductive health term inclusion score was also created based upon a sum of the following codes (1 if present, 0 if absent): reproductive health, abortion, contraception, and emergency contraception. The correlation between the reproductive health term inclusion score and length of policy was assessed to discern whether mention of terms was more or less likely with the associated length of the policy.

For each hospital – religious affiliation and type of religion affiliation was coded. Bivariate analyses of each policy reproductive health coded term and Catholic hospital affiliation was assessed using the Chi square test statistic and an alpha value of 0.05.

## Results

There were 108 hospitals listed on the Washington State Hospital Association Website [18]. On the Washington State Department of Health public reproductive health policy website [17], 98 (91%) hospitals were listed. Of the 98 hospitals, 88 (90%) hospitals had posted a reproductive health policy. Of the 88 hospitals included, 35% were part of a larger organization. Nearly a third, 30%, were religiously affiliated: 96% Catholic and 4% Seventh-Day-Adventist. Among the Catholic hospitals, 81% were part of a conglomerate.

The policy length ranged from a quarter of a page to 18 pages (mean = 2). The mean term inclusion score for non-Catholic hospitals, 1.6, was significantly less than the score, 2.2, for Catholic hospitals. The correlation between policy length and term inclusion score was (− 0.39) for non-Catholic and (−.41) Catholic affiliated hospitals, suggesting a negative relationship between policy length and inclusion of reproductive health terms.

### Reproductive health care theme

Just 48% of the non-Catholic hospital’s policies mentioned reproductive health compared to 73% of the Catholic hospitals, a significant difference (see Table 1). When policies did include mention of reproductive health, the statements were often vague.

**Table 1:** Washington State Hospital Reproductive Health Policies Term and Information Inculsion, by Catholic Affiliation, 2016

Reproductive Health*	
Catholic	73%
non-Catholic	48%
Legal Language	
Reproductive Health in terms of Legal Language*	
Catholic	0%
non-Catholic	18%
Abortion in terms of Legal Language*	
Catholic	0%
non-Catholic	24%
Contraception Service Provision in terms of Legal Language	
Catholic	0%
non-Catholic	3%
Emergency Contraception Service Provision in terms of Legal Language	
Catholic	19%
non-Catholic	10%
Abortion	
Catholic	58%
non-Catholic	60%
Abortion Provision	
Catholic	39%
non-Catholic	40%
Abortion Type*	
Catholic	
medical	39%
medical & elective	0%
referral	0%
unclear	0%
non-Catholic	
medical	11%
medical & elective	13%
referral	5%
unclear	16%
Protecting the Provider*	
Catholic	0%
non-Catholic	44%
Contraceptive Services	
Catholic	35%
non-Catholic	31%
Contraception Service Provision	
Catholic	19%
non-Catholic	16%
Contraception Service Provision Clarity	
Catholic	4%
non-Catholic	6%
Emergency Contraception*	
Catholic	54%
non-Catholic	21%
Emergency Contraception Only for Sexual Assault*	
Catholic	54%
non-Catholic	16%

*p < 0.05

“The purpose of this policy is to provide our patients with a clear summary of the commitments and expectations in these subject areas.” This same hospital included this:

“… to provide access to female and male reproductive healthcare services to meet a patient’s clinical needs and a patient’s choice, although not every procedure is available.” (Non-Catholic, Independent).

Among hospital policies that did not include any mention of reproductive health, some examples include: a general “Patient’s Rights and Responsibilities” pamphlet and a 7-page forensic nurse procedure for domestic violence cases.

### Reproductive health, abortion, contraception, and emergency contraception service provision in terms of the law

Non-Catholic hospital polices wrote about reproductive health (18%), abortion (24%), and contraceptive service provision (3%) in terms of legalese while none of the Catholic hospitals did, a significant difference by Catholic affiliation for both reproductive health and abortion. For emergency contraception, Catholic hospitals used legal language (19%) more so than non-Catholic hospitals (10%), but this difference is not statistically significant (see Table 1).

“It is the policy of Jefferson Healthcare to abide by RCW’s 0.02.100^1^ and 9.02.160^2^ within the limitations of the resources and services offered at the organization.” (Non-Catholic, Independent).

Without an understanding of those laws – which take time and effort to locate online, this policy would not provide much clarity for the patient about what these hospitals provide in terms of reproductive health care to patients at those hospitals. Additionally, the language of “within the limitations of the resources and services offered” would also require insider information about the resources at that particular facility.

“Sunnyside Community Hospital will provide information to female patients in accordance with the requirements of RCW 9.02.160^2^ when requested by the patient.” (Non-Catholic, Independent).

A patient at this hospital would need to know about the policy in order to know what to ask, regardless, the result might be a referral to another location.

Some hospital policies would state the law, then state they didn’t provide any maternity care services.

“The public hospital district complies with the Reproductive Privacy Act….The public hospital district does not provide maternity care benefits, services, or information or pregnancy termination benefits, services or information.” (Non-Catholic, Independent).

The law states that provision of maternity services as a precondition for providing reproductive health services including contraceptive and abortion services. This policy states that the hospital complies with the law, since they do not offer maternity services, they do not provide contraception and abortion services.

### Abortion, abortion provision, and abortion type

Very few policies explicitly named abortion. A similar percentage of non-Catholic and Catholic policies include the abortion theme (58–60%) and abortion provision (39–40%) – not statistically significant differences. Among the non-Catholic sample, 13% provide medically-indicated and elective abortions, 11% provide medically-indicated only, 5% refer out, and for 16% provision of abortion types was not stated or was unclear. All Catholic policies that included abortion provision (39%) specified only medically-indicated abortions were provided, these differences were statistically significant (see Table 1). The following are examples of clear language in terms of abortion provision:

“Within the UW Medicine integrated health system, we offer both elective and medically indicated terminations of pregnancy...” (Non-Catholic, Conglomerate).

“Elective abortion of normal pregnancy is NOT allowed in Deaconess Hospital.” (Non-Catholic, Independent).

While some hospitals asserted support for women’s right to abortion services, they did not always provide it. For example, one hospital included inclusive introductory language for their policy:

“This policy defines the woman’s right to appropriate health care services that will enable her to go safely through pregnancy and childbirth and the freedom to decide if, when and how often to reproduce.”

Yet, two sentences later this is stated:

“Grays Harbor Community Hospital does not provide medical or elective termination of pregnancies.” (Non-Catholic, Independent).

The following policy used language from the ERDs to describe their policy in terms of abortion provision:

“PeaceHealth does not allow direct abortions. PeaceHealth allows the indirect termination of a pregnancy as a result of direct intervention against a maternal pathology to save the life of the mother.” (Catholic, Conglomerate).

However, for a lay audience, the terms of direct vs. indirect abortion might not be understood – nor what a “direct intervention against a maternal pathology” might involve.

One hospital’s entire policy was about how to handle a non-viable pregnancy. In this policy, the word abortion is never mentioned and termination of pregnancy is only mentioned once. The procedure is most often referred to as labor (Catholic, Independent).

Another hospital referred to offering termination of pregnancy due to the state law but later in the policy there were many barriers to care noted as outlined in the “pretermination” period. These processes involved at least six individuals from the hospital, paperwork on behalf of the patient and the provider, a minimum waiting period of 48 h, and a social service referral (Non-Catholic, Independent).

Another hospital clearly stated they only provide medically-indicated abortions; however, the process for providing medically-indicated abortions involved (11) hospital personnel and (13) hospital procedures (Non-Catholic, Independent).

The quote below is the entire reproductive health policy for this hospital:

“Termination of pregnancy for fetal anomalies/genetic condition is available at Yakima Valley Memorial Hospital for those patients with a confirmed diagnosis who have received genetic counseling and are making an informed decision. That is the only case in which termination is available.” (Non-Catholic, Independent).

In 5% of the non-Catholic hospitals referral out for abortion was emphasized. In this example, the policy indicates they follow state policies about providing pregnancy terminations, but the language implied they would do so only through referral.

“Referral and informational services are provided to offer women and family choices regarding voluntary termination of pregnancy.” (Non-Catholic, Independent).

In one hospital policy, a detailed section on abortion begins with language about providing information and transfer and ends with language about sanctions against providers who provide abortions, this is an excerpt from the following:

“All providers at Lincoln Hospital District #3 are expected to respond to any patient’s questions about birth control and pregnancy-terminating procedures with openness and compassion....Any female patient wishing to receive pregnancy-terminating medication (excluding emergency contraception) or medical procedures while a patient at this hospital will be assisted in transfer to another facility...If a provider participates in termination procedures beyond what is allowed by hospital policy, Lincoln Hospital District #3 may impose sanctions on that provider.” (Non-Catholic, Independent).

### Protecting the provider against forced abortion provision

Only non-Catholic hospital policies (44%) included details on health providers’ freedom to opt out of providing care, a statistically significant difference (see Table 1). It was common to read a non-Catholic policy that first stated the law, and then included the opt-out provision for providers, as this example shows:

“The Public Hospital District complies with the Reproductive Privacy Act…The Act contains a conscience clause acknowledging that no person may be required in any circumstance to participate in the performance of an abortion if such person objects to doing so. The Public Hospital District respects each individual’s right to refuse to participate in an abortion if such person objects to doing so.” (Non-Catholic, Independent).

One hospital’s entire policy was about the provider’s right to opt out of providing services (Non-Catholic, Independent).

The following hospital policy provides extensive detail regarding the provider opt-out option and notes that if all providers opted-out, it would be impossible to provide abortions at the facility – highlighting the tension between two laws – one that protects patient access to abortion and the other that protects providers from having to provide abortion services:

“Public hospital districts cannot perform abortions without the assistance of their medical staff and employees. The Act prohibits a public hospital district from requiring its medical staff or employees to participate in the performance of abortions. A public hospital district also is prohibited from requiring a physician by contract (including employment) to perform abortions. Physicians who refuse to perform abortions cannot be denied privileges nor can their medical staff privileges be adversely affected. As a result, if Newport Hospital & Health Services medical staff or employees are unwilling to perform abortion services, it may be impossible for a public hospital district to provide abortion services that are substantially equivalent to the maternity care services available at its facilities.” (Non-Catholic, Independent).

### Contraceptive services, provision of contraceptive services, and clarity of contraceptive service provision

A similar percentage of non-Catholic and Catholic policies mention contraceptive services (31–35%), provision of contraceptive services (16–19%), and have clear language about provision (4–6%), none of which differ significantly by hospital Catholic affiliation (see Table 1). The following is an example of clear language in regards to provision of preventative services, including contraception.

“Through the primary care settings in hospital facilities, patients have access to a full array of preventative healthcare services including all forms of contraception prevention, and the prevention and treatment of sexually transmitted diseases.” (Non-Catholic, Conglomerate).

In contrast, this hospital policy provides absolutely no clarity in terms of contraceptive service provision as it is unclear how the hospital responds to the law:

“The Act declares that it is the public policy of the state Washington that every individual has the fundamental right to choose or refuse birth control. The Act does not, however, impose an affirmative duty on the state or its municipal corporations, such as public hospital districts, to provide birth control or other family planning or reproductive” (Non-Catholic, Independent).

### Emergency contraception

Emergency contraception (EC) was mentioned in 21%, and only in terms of sexual assault in 16%, of the non-Catholic hospital policies, while it was mentioned in 54% of Catholic policies, all of which made reference to sexual assault, both of which are significantly different by Catholic affiliation (see Table 1).

“Forks Community Hospital provides emergency contraception to victims of sexual assault only.” (Non-Catholic, Independent).

Stating clearly that should a patient who doesn’t indicate sexual assault request emergency contraception, they will be denied those services.

The following is the first sentence of this hospital’s reproductive health policy and states that the purpose of emergency contraception is only to prevent pregnancy following sexual assault, this is just one example of a few policies making this incorrect statement:

“The purpose of PCC (post-coital contraception) is to prevent pregnancy following a sexual assault.” (Non-Catholic, Independent).

This policy continues to outline the guidelines and procedures for administering emergency contraception to sexual assault victims. The entire focus of the guidelines is to determine among the population of sexual assault victims, which ones should receive emergency contraception.

Another hospital only included emergency contraception in their posted reproductive health policy. They indicated that emergency contraception information would be provided to both victims of sexual assault and those who had unprotected sex; however, only victims of sexual assault would receive the actual provision of emergency contraception (Non-Catholic, Independent).

## Discussion

This research examined the content regarding abortion and contraception service provision among the posted reproductive health policies of Washington State hospitals, by hospital Catholic affiliation, in response to a call to study religious and non-religious hospital reproductive health policy transparency [11, 19]. In general, a lack of clarity about abortion and contraception service provision existed in the reproductive health policies posted by the hospitals in Washington State, regardless of Catholic affiliation, despite some of the language used to assert clarity. Overall, there were more similarities than differences between the non-Catholic and Catholic hospital reproductive health policies. The Catholic hospital policies were more likely to include reproductive health terms than the non-Catholic hospitals; however, they were less likely to indicate provision of reproductive health care – even among those areas of care mentioned. In sum, all hospital reproductive health policies, regardless of religious affiliation, lacked transparency about abortion and contraception service provision. In addition, the length of the policy was negatively correlated with inclusion of the main reproductive health terms of focus in this study.

Not all reproductive health policies included a mention of reproductive health and a few policies were policies about not providing reproductive health care. Three percent to a quarter of the non-Catholic polies were written using the language of existing laws – whether in general or for specific issues. Nearly half of the non-Catholic reproductive health policies included a provision about provider’s choice to opt-out of providing abortion care. None of the Catholic hospitals included a mention of provider opt-out, likely as it is a non-issue for facilities not performing abortions.

The most common subject included in the non-Catholic policies was abortion – yet, the term abortion was rarely used. Despite the common inclusion of the theme of abortion, just 24% clearly indicated the type of abortion provided. Emergency contraception was included in about a fifth of the non-Catholic policies. In the Catholic policies, 54% mention emergency contraception – and all were in relation to its use for sexual assault victims exclusively. In sum, the impetus for requiring hospitals in Washington State to publicly post their reproductive health policies to increase transparency in provision of abortion and contraception services was not met based upon the facts that the mere mention of important terms/themes never met 100% (21–73%) and the inclusion of details about provision of care was even lower (4–40%).

Reproductive health care is vast and encompasses many aspects of medical care. A policy on all types of reproductive health services provided by a hospital facility would be lengthy. As a result of this issue and the desire to increase transparency in types of reproductive care provided – standardized checklists including reproductive health care that differs from the standard of care is what should be included in a transparent reproductive health policy. Reproductive health procedures that potentially fall into this category include: abortion, contraception, sterilization, emergency contraception, ectopic pregnancy, and LGBTQI care – as well as others. For each of these categories, the checklist should make it clear what the organization does and does not provide [19] – especially as it differs from the medical guidelines [6]. See Table 2 as a recommended checklist template for abortion and contraceptive service provision for hospitals.

**Table 2:** Abortion and Contraception Service Provision Checklist for Hospitals

Services Provided and Available to all Patients 24/7	YES	REFERRAL	NO	UNSURE	Comments
Abortion Provision					
Medically-indicated abortions					
Elective abortions					
Contraceptive Method Provision, for Any Reason, Including Pregnancy Prevention					
Modern Methods (condoms, pill, injectable, implant, IUD)					
Sterilization (vasectomy and tubal ligation)					
Emergency Contraception, for victims of sexual assault					
Emergency Contraception, irrespective of sexual assault					

If providers are uncomfortable with a procedure, they do not have to provide it. Due to this right, if a patient needs services but all of the providers opt-out of providing the service, at this time the patient can’t receive that service at that facility. Hospitals, and providers, are protected from discomfort in terms of abortion provision; however, there is an absence of protection for the individual patient in need of care [20]. Provider protections to not provide services have recently gained momentum. In 2018, the federal department of Health and Human Services created a Conscientious and Religious Freedom Division as well as released a new rule to strengthen provider’s ability to refuse to provide services that are against their beliefs. The World Health Organization notes that providers are allowed conscientious objection to abortion provision – but that they have a duty to refer those patients to a provider, or facility, that will provide those services and in the absence of those options, and if it is not possible to ensure a referral that the provider must provide the abortion to avoid undue harm to the patient [21].

“Americans should be able to count on receiving care that meets their needs and is based on the best scientific knowledge--yet there is strong evidence that this frequently is not the case. Health care harms patients too frequently and routinely fails to deliver its potential benefits.” [20]. The Institute of Medicine highlights that care should be safe, patient-centered, and timely as core needs for health care: “The patient is the source of control. Patients should be given the necessary information and opportunity to exercise the degree of control they choose over health care decisions that affect them. The system should make available to patients and their families information that enables them to make informed decisions…” [20]. It is clear that these ideals are currently not being met in abortion and contraceptive care at hospitals in Washington State, regardless of religious affiliation.

Research has shown that hospital reproductive health policies are often unclear to those providing the services as well [22]. Therefore, hospitals publicly posting transparent abortion and contraceptive service standardized checklists is just one necessary step for both providers and patients– but so is abundant patient education and awareness at the population level, during patient encounters, and in a pre-emptive manner by hospitals.

Throughout this research it was clear that some policies were really unique, and often avoided addressing all, or any, abortion and contraceptive service provision policy, while others were notable only for their similarity to the others – while remaining ambiguous. This pattern observed makes one wonder about the reach of religious healthcare on other healthcare – as it is clear that institutions look to each other for guidance.

The limitations of this research are that the content analysis of the reproductive health policies was limited to the posted reproductive health policies. Efforts were not made to find clarification to the policies by further research into the hospitals themselves. Catholic affiliation was dichotomized but the relationship between Catholic affiliation and hospital care falls along a spectrum. There were aspects of reproductive health in the hospital polices that were not included in the analysis, most often due to lack of attention to the issue, some examples include: ectopic pregnancy, sterilization, labor and delivery, sexually transmitted infections, infertility, genetic testing, and domestic violence.

Despite the limitations, this research did have a few strengths. Three researchers collaborated to create the content analysis plan. Coding was implemented independently by all three researchers – and all issues that arose during the analysis were discussed by all three and resolved in an agreeable manner. Additionally, the quantitative findings were illustrated through qualitative selection of illustrative quotes.

Future research should continue to find reasons for restrictions on abortion and contraceptive service provision at non-religious hospitals as well as the best ways to educate the public about these restrictions. It would also be interesting to know how this knowledge affects care-seeking behavior; however, it is also true that various options for care are not available to everyone.

## Conclusion

In sum, the current reproductive health policies among Washington State hospitals rarely provide clarity in terms of abortion and contraception service provision for the people of Washington State – regardless of Catholic affiliation of the hospital. Public posting of a comprehensive reproductive health policy would not achieve clarity given the breadth of hospital reproductive health services. As a result, the best way to increase transparency about abortion and contraceptive service provision policy would be to require hospitals to publically post responses to a standardized checklist (see Table 2). More efforts are needed to educate the public, generally, and in patient and provider encounters about abortion and contraception service provision options. The same level of protection provided for hospitals and providers to not offer abortion and contraceptive services needs to be afforded to individual patients in the arena of receiving abortion and contraception services. It is vital that patient rights to information about contraception and abortion service provision, at all health facilities offering reproductive healthcare, regardless of religious affiliation, be rectified immediately.

## Footnotes

1 The legal language of RCW 9.02.100 is as follows: The sovereign people hereby declare that every individual possesses a fundamental right of privacy with respect to personal reproductive decisions. Accordingly, it is the public policy of the state of Washington that: (1) Every individual has the fundamental right to choose or refuse birth control; (2) Every woman has the fundamental right to choose or refuse to have an abortion, except as specifically limited by RCW 9.02.100 through 9.02.170 and 9.02.900 through 9.02.902; (3) Except as specifically permitted by RCW 9.02.100 through 9.02.170 and 9.02.900 through 9.02.902, the state shall not deny or interfere with a woman’s fundamental right to choose or refuse to have an abortion; and (4) The state shall not discriminate against the exercise of these rights in the regulation or provision of benefits, facilities, services, or information.

2 The legal language of RCW 9.02.160 is as follows: If the state provides, directly or by contract, maternity care benefits, services, or information to women through any program administered or funded in whole or in part by the state, the state shall also provide women otherwise eligible for any such program with substantially equivalent benefits, services, or information to permit them to voluntarily terminate their pregnancies.
